# Interaction Effect of Phase Angle and Age on Femoral Neck Bone Mineral Density in Patients with Non-Dialysis Chronic Kidney Disease Stage 5

**DOI:** 10.3390/nu15071680

**Published:** 2023-03-30

**Authors:** Byoung-Geun Han, Daewoo Pak, Jun Young Lee, Jae-Seok Kim, Jae-Won Yang, Seongyup Kim

**Affiliations:** 1 Department of Nephrology, Yonsei University Wonju College of Medicine, Wonju 26426, Kang-won, Republic of Korea; 2 Division of Data Science, Yonsei University, Wonju 26493, Kang-won, Republic of Korea; 3 Department of Surgery, Yonsei University Wonju College of Medicine, Wonju 26426, Kang-won, Republic of Korea

**Keywords:** aging, body composition, bone mineral density, chronic kidney disease, impedance

## Abstract

***Background:*** Low bone mass is common in malnourished patients with chronic kidney disease (CKD) and can lead to a higher risk of fractures. Elderly and CKD patients have the same risk factors for protein-energy wasting, sarcopenia, and osteoporosis. Here, we investigated the association between phase angle (PhA) and bone mineral density (BMD) in dialysis-naïve patients with CKD stage 5 (CKD5) and identified a statistical relationship between PhA and age, which affects bone density. ***Methods:*** Bio-impedance spectroscopy for evaluating body composition and PhA and dual-energy X-ray absorptiometry for determining the BMD were simultaneously performed in 167 consecutive patients (mean age, 59.65 ± 13.98 years; women, 40.1%). Two-way analysis of variance (ANOVA) was conducted to assess the potential interaction effect of PhA and age on femoral neck BMD (FN-BMD). ***Results:*** Our results showed that PhA and age were independently associated with FN-BMD and T-score in multiple linear regressions analyses. A significant interaction effect of PhA and age on FN-BMD was found on two-way ANOVA (*p* = 0.028). The average BMD values for the first and second tertiles of the PhA were higher in the young versus elderly group, whereas patients in the elderly group had higher BMD in the third tertiles. ***Conclusions:*** A relationship was noted between PhA and BMD in patients with advanced-stage CKD. The effect of PhA level on FN-BMD differed between elderly and young patients. Our study suggested that higher PhA levels could be a marker explaining the maintenance of good bone health in elderly patients with CKD5. Further longitudinal analyses are needed to determine whether PhA predicts the risk of CKD-MBD-related fractures during CKD progression.

## 1. Introduction

As the risk of fracture increases with age in the general population, patients with chronic kidney disease (CKD) develop significantly higher fracture rates than age- and sex-matched individuals without CKD [1]. Low bone mass is common in patients with reduced renal function for many reasons, in addition to age-related and postmenopausal bone loss. Although CKD has been proposed as a model for early bone aging, the concept of osteoporosis in the general population cannot fully explain the bone status of patients with CKD. Patients with CKD and end-stage renal disease (ESRD) may have bone mineral disorders related to CKD, making the diagnosis of osteoporosis relatively difficult and highly complex, even with dual-energy X-ray absorptiometry (DEXA). No non-invasive imaging or biomarker tests can accurately reflect bone turnover and mineralization in this patient group. Nevertheless, the Work Group of Kidney Disease: Improving Global Outcomes recommends DEXA for evaluating the bone status of patients with CKD as well as the general population [2].

Bone density has traditionally been reported as significantly lower in malnourished patients with chronic diseases. As CKD progresses, protein-energy wasting (PEW) can lead to changes in body composition that may affect bone health. Although studies have shown significant relationships between body composition and bone mineral density (BMD), inconsistent results have been reported for various factors such as age, sex, and specific diseases [3,4]. Most previous studies analyzed patients undergoing dialysis [5,6,7]. However, few studies have investigated the relationship between body composition and bone density in patients with non-dialysis CKD.

The impedance-derived phase angle (PhA) is an indicator of cell membrane integrity and physiological functions of cells [8]. PhA is a raw bio-impedance parameter without assumptions; therefore, it is less biased and can be applied for comparisons in several disease conditions. In healthy adults, age, sex, and body mass index (BMI) are major determinants of PhA [9]. PhA is also associated with decreased muscle mass and strength, nutritional status, disease prognosis, and mortality in many clinical situations [10,11,12,13,14,15]. PhA, a useful marker of nutritional status in patients with ESRD, is significantly associated with PEW [16]. Malnutrition, particularly protein deficiency, contributes to the development of osteoporotic fractures by altering muscle function and reducing bone mass [17,18]. However, few studies have applied PhA to estimate the association with BMD [15,19,20,21]. Furthermore, few studies have statistically analyzed the relationship between PhA and age, which affects bone density in patients with CKD.

Elderly individuals and patients with CKD are thought to have the same risk factors for combination diseases, such as PEW, sarcopenia, and osteoporosis. Therefore, here, we investigated whether PhA is associated with BMD in dialysis-naïve patients with stage 5 CKD (CKD5), and subsequently identified a potential interaction effect of PhA and age on femoral neck BMD.

## 2. Materials and Methods

### 2.1. Patients and Data Collection

Since 2014, consecutive patients with CKD5 have been registered in this bio-impedance cohort. All patients were hospitalized to plan their first dialysis treatments, and all underwent bio-impedance spectroscopy (BIS) and laboratory evaluations, at the time of enrollment, prior to the initiation of dialysis. Of the total patient cohorts, 167 were simultaneously evaluated for BMD. Therefore, the current retrospective observational study analyzed a prospective cohort database of dialysis-naïve patients with CKD5 without non-traumatic bone fractures. The study was conducted in accordance with the principles of Declaration of Helsinki. The study was initiated after obtaining approval (no. CR316024) approved by the Institutional Review Board of Yonsei University Wonju Severance Christian Hospital. All patients provided written informed consent before participating in the study.

### 2.2. BMD Assessment

BMD was measured using the DEXA method (Horizon W; Hologic, Inc., Marlborough, MA, USA) at the femoral neck and lumbar spine (L1–L4) using an anteroposterior projection. The absolute BMD values (g/cm^2^) and T-scores for each region were reported. The trabecular bone score (TBS) was obtained from lumbar spine DEXA 2-D images using TBS iNsight Software^®^ (version 2.1.2.0; Medimaps, Merignac, France).

### 2.3. Body Composition Measurements

Whole-body BIS was performed with patients in the supine position using the Body Composition Monitoring^TM^ (BCM) system (Fresenius Medical Care, Bad Homburg vor der Höhe, Germany) prior to any dialysis treatment. The BCM system utilizes alternating electric currents across 50 discrete frequencies covering the 5–1000 kHz frequency spectrum and measures the impedance of each current. Total body, extracellular, and intracellular water were calculated automatically. The overhydration (OH, L) value was also provided as a marker of fluid balance. The accuracy of BCM-measured OH has been reported [22]. Therefore, the patient’s BMI was recalculated by considering fluid overload using the following formula: corrected BMI (cBMI, kg/m^2^) = (body weight-OH)/height^2^. PhA is the angle of the time delay between the voltage waveform at 50 kHz and the current waveform. PhA is directly calculated from the electrical components of resistance (R) and reactance (Xc) and is converted from radians to degrees as the arc tangent of the reactance-to-resistance ratio. A close correlation between nutritional status and PhA has been demonstrated in several studies [23]; therefore, PhA was used as a marker of nutritional status in our study.

### 2.4. Statistical Analysis

The patients’ demographic and clinical characteristics are summarized as frequencies and percentages for categorical variables and means with standard deviations for continuous variables. The assumption of normality was examined using the Kolmogorov–Smirnov test and normal QQ-plots. All patients were divided into three groups based on the tertiles of femoral neck BMD and PhA for analysis. The distributions of the patient characteristics across the tertiles of femoral neck BMD were compared using a chi-squared test, and the differences in means were tested using a one-way analysis of variance (ANOVA) with a post hoc Tukey honestly significant differences (HSD) test. A linear-by-linear association method and the Jonckheere–Terpstra trend test were used to examine trends in femoral neck BMD tertiles. The association between femoral neck BMD and other variables was evaluated using Pearson’s correlation coefficients. The effects of patient characteristics on the femoral neck BMD were assessed using uni- and multivariate linear regression analyses. Collinearity between variables was determined by examining the variance inflation factor (VIF) or tolerance. Variables with a VIF ≥ 10.0 or tolerance < 0.1 were not included in the multivariable analysis. Variables with values of *p <* 0.10 in the univariate analysis were considered potential determinants for the initial multiple linear regression; next, backward elimination was applied until all remaining variables were significant with values of *p <* 0.05. The Durbin–Watson test was performed to diagnose the autocorrelation of this model. In addition, two-way ANOVA was performed to assess the interaction effect of PhA levels and the two age groups (young and elderly) on the femoral neck BMD or its T-scores. A total of 1000 bootstrap samples were repeatedly extracted to estimate significance.

All analyses were performed using SPSS Statistics software (version 25.0; IBM Corporation, Armonk, NY, USA). Graphs were generated using Prism software (version 5.02; GraphPad Software, San Diego, CA, USA). Statistical significance was defined with *p* < 0.05.

## 3. Results

### 3.1. Patient Characteristics

**Figure 1:**
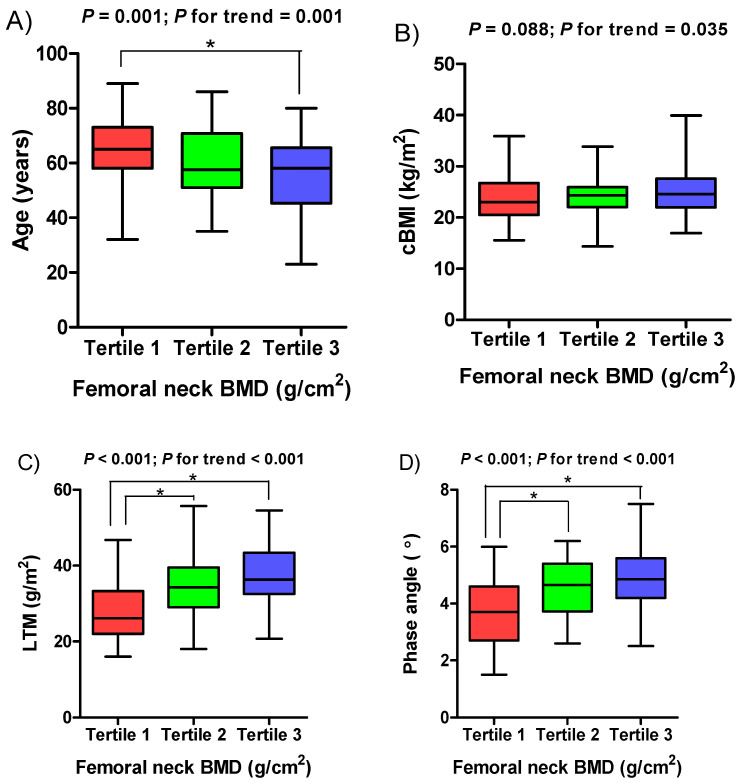
Boxplots of (**A**) age, (**B**) cBMI, (**C**) LTM, and (**D**) phase angle according to femoral neck BMD tertile. BMD, bone mineral density; cBMI, corrected body mass index; LTM, lean tissue mass.

The mean femoral neck BMD and T-score were 0.64 ± 0.14 g/cm^2^ and −1.59 ± 1.09, respectively. The mean PhA was 4.38 ± 1.19°. The observed values of both femoral neck BMD and PhA were normally distributed (Kolmogorov–Smirnov test, *p* = 0.200). The mean ages of the male and female patients were 59.62 ± 13.82 years and 59.69 ± 14.31 years, respectively. Men accounted for 59.9% (*n* = 100) of the patients. Mean femoral neck BMD (0.69 ± 0.12 vs. 0.58 ± 0.14 g/cm^2^, *p* < 0.001), femoral neck BMD T-score (−1.25 ± 0.80 vs. −2.09 ± 1.26, *p* < 0.001), and PhA (4.67 ± 1.11 vs. 3.95 ± 1.17°, *p* < 0.001) values were significantly higher in men. The mean PhA (4.81 ± 1.26 vs. 4.13 ± 1.08°, *p* < 0.001) was significantly lower in patients with diabetes versus those without diabetes, while the femoral neck BMD, T-score, and age were not. The clinical characteristics of patients in the three femoral neck BMD tertiles are presented in Table 1. Patients in the third tertile were significantly younger than those in the first and second tertiles. The second and third tertiles had a significantly higher PhA and lean tissue mass (LTM) than the first tertile. There were increasing trends in LTM and PhA in the femoral neck BMD tertiles, whereas age showed a substantial decreasing trend (Figure 1). The cBMI tended to increase significantly as the tertile of the femoral neck BMD increased, although no significant difference was detected among the three groups. Serum biochemical parameters were not significant different among the three groups. In contrast, serum alkaline phosphatase (ALP) and intact parathyroid hormone (iPTH) levels were marginally significant, with *p* values close to 0.05.

### 3.2. Correlation of Body Composition and Laboratory Parameters with Femoral Neck BMD and T-Score

Femoral neck BMD and T-score were positively associated with cBMI, PhA, and LTM, and negatively associated with age and ALP. However, no significant association was found between bone density status and serum markers of mineral metabolism such as intact parathyroid hormone, vitamin D3, and serum calcium and phosphate levels (Table 2).

### 3.3. Multiple Linear Regression Analyses

Multiple linear regression analysis with backward elimination showed that age, serum iPTH, serum ALP, LTM, and PhA were significantly associated with the femoral neck BMD and T-score. The standardized coefficient of PhA was higher than those of the other variables (Table 3). Even when the femoral neck BMD T-score was used as the dependent variable, it was higher than the other values, except for the LTM (Supplementary Table S1).

### 3.4. Two-Way ANOVA

**Figure 2:**
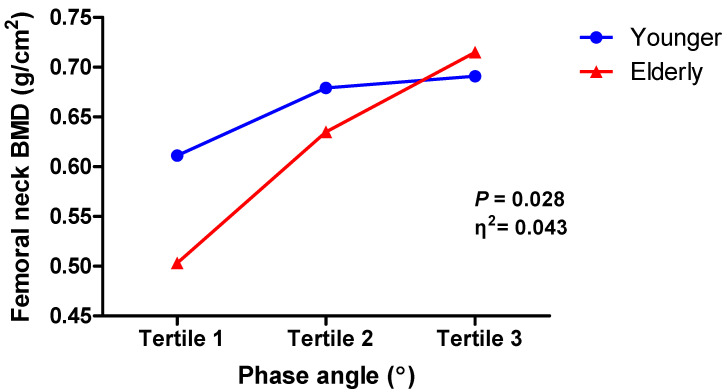
Interaction between phase angle and femoral neck bone mineral density by age. BMD, bone mineral density.

The two-way ANOVA with the interaction effect between PhA and age was performed after dividing the PhA levels into three tertiles (<3.82° for tertile 1; ≥3.82 to <4.98° for tertile 2; and ≥4.98° for tertile 3). Patients’ age was also dichotomized into two group (<65 years for young group and ≥65 years for elderly group). The interaction effect between PhA level and age group was statistically significant (F(2, 161) = 3.66, *p* = 0.028) (Table 4), implying that the effect of PhA levels on femoral neck BMD could differ by age (Figure 2). Bootstrapped 95% confidence intervals did not include “0”. Similar results were found with the femoral neck BMD T-score as a dependent variable, which showed a significant interaction effect between PhA level and age group (F(2, 161) = 5.55, *p* = 0.005) (Supplementary Table S2). The elderly group had a lower mean femoral neck BMD in tertiles 1 and 2 of PhA than the younger group and a higher BMD in tertile 3. Based on the Tukey HSD test, there was a difference in means between PhA tertiles 1 and 2 for the elderly group but not for the younger group, while the means between tertiles 1 and 3 were significantly different for both groups. The mean differences between tertiles 2 and 3 were marginally significant in the elderly group (*p* = 0.08) (Table 5). Post hoc tests for the average of femoral neck BMD T-scores showed similar results (Supplementary Table S3).

## 4. Discussion

Generally, a low body weight is a risk factor for bone loss and fractures. However, the effects of body weight and BMI on BMD have not been fully elucidated [24,25]. Body weight is primarily composed of fat and lean masses. Furthermore, BMI does not reliably account for body composition. The relative impact of lean and fat masses on BMD have been extensively studied [26,27]. However, it has not yet been established which of the two body components is more strongly related [28].

Muscle mass is significantly associated with BMD and could be considered an independent risk factor for a low BMD in the general population and patients undergoing dialysis [7,29,30,31]. A healthy twin study reported that BMD was positively correlated with fat mass and lean mass, but the association was stronger with the latter [32]. On the other hand, fat mass was significantly associated with bone quality in patients on peritoneal dialysis [5,6]. Muscle wasting is common in patients with CKD and closely associated with PEW. PEW is recognized as a major contributor to cardiovascular diseases, osteoporosis, sarcopenia, and frailty. This relationship is multifactorial and complex as hemodynamic changes, hormonal changes, chronic inflammation, and fluid overload progressively worsen with decreased renal function.

Body composition estimates, such as fat mass and fat-free mass from the bio-impedance data are calculated using empirical equations. The BIS can provide raw parameters, such as resistance and reactance, as well as PhA. PhA can be a valid comparison tool for different population groups because it is a direct measure that is unaffected by prediction model assumptions for various body compartments. Higher PhA values indicate greater cellularity, whereas lower values indicate poorer cell health and function [9,14]. PhA decrease with age and can be associated with sarcopenia [10,33]. Previous studies reported that the degree of PhA decrease differs depending on the nature of the causative disease of sarcopenia and that these disease-specific differences may be influenced by age, disease severity, or other confounding factors [14]. Age is an important variable that explains PhA variability in healthy subjects [34,35]. Moreover, age was one of the major determinants for predicting PhA in both healthy and unhealthy people [9,36,37,38]. Furthermore, a new concept in the category of osteosarcopenia was recently introduced, even in patients with non-dialysis dependent CKD [39]. Therefore, to minimize the bias caused by the application of the equations, we investigated the association between PhA, one of the aforementioned raw parameters, and BMD.

Ngai et al. reported that bio-impedance was correlated with BMD using whole-body single frequency (50 kHz) bio-impedance measurements in men and postmenopausal women [40]. Several recent studies investigated the relationship between osteoporosis and PhA [15,19,20,21]. The authors of a large-scale general population study suggested that PhA could be a predictor of osteoporosis unaffected by age and sex [36]. The independent association between PhA and BMD suggests that PhA may have new practical applications for monitoring bone health in the elderly population [18]. Interestingly, other types of crude bio-impedance variables, including height-adjusted resistance (R/H), and height-adjusted reactance (Xc/H), are also related to BMD in the elderly population [37]. Considering that the PhA is calculated from the bio-impedance electrical components of resistance (R) and reactance (Xc), the two previously mentioned studies commonly referred to the new biological implications of PhA [18,37]. The results of a study of 260 women with rheumatoid arthritis (RA) suggested that PhA could serve as a potential predictor of RA prognosis and the concomitant development of osteoporosis. PhA is significantly associated with spongial BMD and cortical index in patients with RA [21]. Similar to these previous reports, our results demonstrated a positive correlation between PhA and femoral neck BMD. Femoral neck BMD was used in this study because calcifications of the abdominal aorta can lead to overestimation of lumbar spine BMD. As changes in estrogen levels are key drivers of bone loss in postmenopausal women [41], numerous hormonal and metabolic disturbances play major roles in the development of renal osteodystrophy in patients with advanced-stage CKD [42]. These changes are further aggravated by aging. With age, alterations in body composition and additional hormonal imbalances, albeit part of the pathophysiology, may result in changes in BMD. If changes in body composition induced by renal function deterioration or aging were viewed from a collinear perspective, the two pathological conditions in our study may have affected PhA alone or in combination. Therefore, it is necessary to fully consider the meaning inherent in age when analyzing the clinical significance of the PhA.

Age may be independently associated with PhA variability. Therefore, in the regression analyses of many previous studies, age was often used as a covariant. Despite this, the interplay between age and PhA may coexist as a marker of the development and deterioration of cachexia, sarcopenia, and frailty. However, few studies have conducted three-dimensional analyses of the relationship between two variables (age and PhA) and bone density in patients with CKD. In our study, we examined the status of femoral neck BMD according to PhA distribution by age (Figure 2). Thus, we were able to confirm an interaction effect between age and PhA on femoral neck BMD.

This study had some limitations. First, the number of patients participating in the analysis was relatively small, as was the proportion of women versus men. We could not determine whether this had an effect on the analytical results. Second, we did not measure the above-mentioned parameters across all CKD stages. Therefore, the statistical significance of two-way ANOVA on whether there was an interaction effect could not be determined for other CKD stages. Third, the association between nutritional markers and bone mass cannot be fully determined using a single BIS or DEXA measurement. However, the results of this study suggest that a longitudinal study is required to examine the relationship between PhA and BMD. Fourth, since no bone biopsy was performed, our study findings cannot represent the entire bone status. The histopathological term “renal osteodystrophy” is a simple classification of bone morphology, while the clinical term “chronic kidney disease-mineral bone disorder (CKD-MBD)” focuses on information regarding overall bone metabolism. DEXA does not reflect the histopathological changes or bone mineral metabolism. Finally, there may have been unmeasured additional variables associated with BMD and muscle strength because our patients had complicated combinations of PEW, increased uremic toxins, systemic inflammation, hemodynamic instability, hormonal changes, and administration of numerous drugs. These factors were not sufficiently considered due to the limitations of the research design. To the best of our knowledge, despite these limitations, the strength of our study is that this is the first to attempt such an analysis.

## 5. Conclusions

Here, we identified a relationship between PhA and BMD in patients with advanced-stage CKD. Our results suggest that PhA could be a biomarker of BMD in these patients. Further longitudinal analyses may determine whether PhA predicts the risk of CKD-MBD-related fractures during CKD progression. Two-way AVOVA showed that the effect of PhA level on femoral neck BMD was different in elderly versus younger patients. Along with the use of these statistical techniques, the practical implications of age should be considered when interpreting the clinical significance of PhA.

## Figures and Tables

**Table 1:** Patient demographics, body compositions, and serum chemistry according to femoral neck bone mineral density tertile.

Variable	Total (*n* = 167)	Femoral Neck Bone Mineral Density			*p* Value (*p* for Trend)
		Tertile 1 (*n* = 55)	Tertile 2 (*n* = 56)	Tertile 3 (*n* = 56)	
Age, years	59.65 ± 13.98	64.44 ± 13.70	59.66 ± 12.43	54.93 ± 14.32 ^b,c^	0.001 (0.001)
≤65, years	107 (64.1%)	28 (26.2%)	37 (34.6%)	42 (39.3%)	0.028 (0.008)
>65, years	60 (35.9%)	27 (45.0%)	19 (31.7%)	14 (23.3%)	
Sex					
Male	100 (59.9%)	17 (17.0%)	41 (41.0%)	42 (42.0%)	<0.001 (<0.001)
Female	67 (40.1%)	38 (56.7%)	15 (22.4%)	14 (20.9%)	
Phase angle, °	4.38 ± 1.19	3.68 ± 1.15	4.61 ± 0.98 ^a^	4.86 ± 1.10 ^b^	<0.001 (<0.001)
Diabetes					
Yes	105 (62.9%)	35 (33.3%)	36 (34.3%)	34 (32.4%)	0.917 (0.750)
No	62 (37.1%)	20 (32.3%)	20 (32.3%)	22 (35.5%)	
FN-BMD, g/cm^2^	0.64 ± 0.13	0.50 ± 0.10	0.64 ± 0.03 ^a^	0.78 ± 0.07 ^b,c^	<0.001 (<0.001)
FN-BMD T-score	−1.59 ± 1.09	−2.75 ± 0.71	−1.60 ± 0.28 ^a^	−0.44 ± 0.56 ^b,c^	<0.001 (<0.001)
LS-BMD, g/cm^2^	0.96 ± 0.18	0.85 ± 0.14	0.97 ± 0.15 ^a^	1.07 ± 0.18 ^b,c^	<0.001 (<0.001)
LS-BMD T-score	−0.47 ± 1.52	−1.44 ± 1.13	−0.41 ± 1.24 ^a^	0.41 ± 1.52 ^b,c^	<0.001 (<0.001)
TBS, lumbar spine	1.37 ± 0.10	1.32 ± 0.10	1.38 ± 0.08 ^a^	1.42 ± 0.08 ^b^	<0.001 (<0.001)
cBMI, kg/m^2^	24.29 ± 4.05	23.35 ± 4.15	24.48 ± 3.56	25.02 ± 4.30	0.088 (0.035)
LTM, kg	32.87 ± 8.52	27.30 ± 7.22	34.06 ± 7.88 ^a^	37.14 ± 7.42 ^b^	<0.001 (<0.001)
ATM, kg	30.86 ± 11.47	29.92 ± 11.22	30.80 ± 10.04	31.84 ± 13.08	0.680 (0.632)
Ferritin, ng/mL	205.74 ± 222.50	229.10 ± 211.25	208.84 ± 281.53	179.71 ± 158.43	0.503 (0.306)
hs-CRP, mg/dL	0.93 ± 2.47	0.71 ± 1.51	1.27 ± 3.31	0.79 ± 2.24	0.436 (0.763)
iPTH, pg/mL	324.90 ± 241.58	388.15 ± 324.93	293.69 ± 212.24	294.00 ± 146.52	0.059 (0.200)
Vitamin D3, ng/mL	15.19 ± 9.31	14.20 ± 8.11	15.04 ± 10.30	16.31 ± 9.41	0.496 (0.170)
Hemoglobin, g/dL	9.05 ± 1.22	9.05 ± 1.37	8.91 ± 1.14	9.18 ± 1.14	0.508 (0.469)
Total protein, g/dL	6.07 ± 0.73	6.07 ± 0.85	5.99 ± 0.68	6.14 ± 0.67	0.549 (0.486)
Albumin, g/dL	3.54 ±0.50	3.52 ± 0.56	3.51 ± 0.51	3.60 ± 0.42	0.598 (0.365)
Alkaline phosphatase, U/L	80.62 ± 36.94	87.16 ± 45.51	83.59 ± 38.65	71.23 ± 21.18	0.057 (0.132)
Calcium, mg/dL	7.74 ± 1.09	7.80 ± 1.14	7.90 ± 1.00	7.53 ± 1.19	0.180 (0.286)
Phosphate, mg/dL	5.98 ± 1.74	5.92 ± 1.68	5.92 ± 1.68	6.11 ± 1.74	0.798 (0.637)

^a^ Significant difference between tertiles 1 and 2; ^b^ significant difference between tertiles 1 and 3, ^c^ significant difference between tertiles 2 and 3. The femoral neck BMD tertiles 1, 2, and 3 corresponded to <0.58, 0.58–0.70, and >0.70, respectively. ATM, adipose tissue mass; cBMI, corrected body mass index; FN-BMD, femoral neck bone mineral density; hs-CRP, high-sensitivity C-reactive protein; iPTH, intact parathyroid hormone; LTM, lean tissue mass; LS-BMD, lumbar spine bone mineral density; TBS, trabecular bone score.

**Table 2:** Degree of linear correlation between variables.

Variables	Femoral Neck BMD		Femoral Neck BMD T-Score	
	Correlation Coefficient	*p* Value	Correlation Coefficient	*p* Value
Age, years	−0.266	0.001	−0.347	<0.001
Phase angle, °	0.477	<0.001	0.463	<0.001
TBS, lumbar spine	0.489	<0.001	0.504	<0.001
LS-BMD, g/cm^2^	0.597	<0.001	0.621	<0.001
LS-BMD T-score	0.593	<0.001	0.620	<0.001
cBMI, kg/m^2^	0.174	0.025	0.215	0.005
LTM, kg	0.466	<0.001	0.482	<0.001
ATM, kg	0.043	0.577	0.120	0.123
Ferritin, ng/mL	−0.094	0.229	−0.115	0.139
hs-CRP, mg/dL	−0.029	0.709	−0.032	0.679
iPTH, pg/mL	−0.116	0.134	−0.110	0.157
Vitamin D3, ng/mL	0.115	0.143	0.097	0.217
Hemoglobin, g/dL	0.067	0.388	0.057	0.469
Total protein, g/dL	0.120	0.123	0.106	0.171
Albumin, g/dL	0.162	0.036	0.124	0.111
Alkaline phosphatase, U/L	−0.161	0.038	−0.184	0.017
Calcium, mg/dL	−0.007	0.929	−0.039	0.616
Phosphate, mg/dL	0.006	0.934	0.083	0.286

ATM, adipose tissue mass; cBMI, corrected body mass index; hs-CRP, high-sensitivity C-reactive protein; iPTH, intact parathyroid hormone; LTM, lean tissue mass; LS-BMD, lumbar spine bone mineral density; TBS, trabecular bone score.

**Table 3:** Factors independently associated with femoral neck BMD as a dependent variable.

	Unstandardized Coefficients		Standardized Coefficients	T	*p* Value
	B (95% CI)	S.E.	Beta		
Age, years	−0.001 (−0.003, 0.000)	0.001	−0.150	−2.285	0.024
iPTH, pg/mL	−7.44 × 10^−5^ (0.000, 0.000)	0.000	−0.133	−2.031	0.044
ALP, U/L	−0.001 (−0.001, 0.000)	0.000	−0.141	−2.169	0.032
LTM, kg	0.004 ((0.002, 0.006)	0.001	0.259	3.503	0.001
Phase angle, °	0.038 (0.022, 0.055)	0.008	0.337	4.550	<0.001

A significant regression equation was found (F(5, 161) = 18.04, *p* < 0.001) with an R^2^ value of 0.359. The Durbin-Watson statistic was 1.634. ALP, alkaline phosphatase; CI, confidence interval; iPTH, intact parathyroid hormone; LTM, lean tissue mass; SE, standard error.

**Table 4:** Results of two-way analysis of variance for the femoral neck bone mineral density.

Source	Sum of Squares	df	Mean Square	F	*p* Value
Phase angle tertile	0.561	2	0.281	19.430	<0.001
Age group	0.068	1	0.068	4.725	0.031
Interaction between phase angle and age	0.106	2	0.053	3.660	0.028
Error	2.325	161	0.014		
Total	72.035	167			

df, degrees of freedom.

**Table 5:** Post hoc tests of mean femoral neck bone mineral density across phase angle tertiles by age group using Tukey’s honestly significant difference procedure.

Age Group	Difference in Levels	Difference in Means	Difference in SE	95% CI	*p* Value
Younger	Tertile 1–Tertile 2	−0.068	0.031	(−0.141, 0.005)	0.072
	Tertile 1–Tertile 3	−0.081	0.029	(−0.150, −0.012)	0.018
	Tertile 2–Tertile 3	−0.013	0.029	(−0.082, 0.056)	0.901
Elderly	Tertile 1–Tertile 2	−0.133	0.034	(−0.214, −0.051)	0.001
	Tertile 1–Tertile 3	−0.212	0.037	(−0.300, −0.123)	0.000
	Tertile 2–Tertile 3	−0.079	0.037	(−0.168, 0.010)	0.089

CI, confidence interval; SE, standard error.

## Data Availability

It is impossible to faithfully anonymize data for public access. Data is available on request to the Institutional Review Board of Yonsei University Wonju Severance Christian Hospital (irb@yonsei.ac.kr).

## Supplementary Materials

The following are available online at https://www.mdpi.com/article/10.3390/nu15071680/s1, Table S1: Factors independently associated with femoral neck BMD T-score as a dependent variable; Table S2: Results of two-way analysis of variance for the femoral neck bone mineral density T-scores; Table S3: Post hoc tests of mean femoral neck bone mineral density T-score across phase angle tertiles by age group performed using Tukey’s honestly significant difference.
