# Supplementary material for: Interaction Effect of Phase Angle and Age on Femoral Neck Bone Mineral Density in Patients with Non-Dialysis Chronic Kidney Disease Stage 5
Source: Nutrients. 2023 Mar 30;15(7):1680. doi: 10.3390/nu15071680 (PMC10096762; doi:10.3390/nu15071680)
Supplement: Supplementary file 1 [file nutrients-15-01680-s001.zip › nutrients-2292526-supplementary.pdf]

Supplementary Table S1. Factors independently associated with femoral neck BMD T-score as a dependent variable.

	Unstandardized Coefficients		Standardized Coefficients	T	*P* value
	B (95% CI)	SE	Beta		
Age, years	-0.017 (-0.027, -0.007)	0.005	-0.217	-3.373	0.001
iPTH, pg/mL	-0.001 (-0.001, 0.000)	0.000	-0.128	-2.008	0.046
ALP, U/L	-0.005 (-0.009, -0.001)	0.002	-0.171	-2.718	0.007
Uric acid, mg/dL	0.046 (-0.008, 0.099)	0.027	0.104	1.682	0.094
LTM, kg	0.041 ((0.021, 0.061)	0.010	0.320	3.982	<0.001
ATM, kg	0.013 (0.000, 0.026)	0.007	0.134	1.914	0.057
Phase angle, °	0.240 (0.101, 0.378)	0.070	0.263	3.424	0.001

A significant regression equation was found ($F(7, 156) = 16.22$, $P < 0.001$) with an R^2 value of 0.421. The Durbin-Watson statistic was 1.685.

ALP, alkaline phosphatase; ATM, adipose tissue mass; CI, confidence interval; iPTH, intact parathyroid hormone; LTM, lean tissue mass; SE, standard error.

Supplementary Table S2. Results of two-way analysis of variance for the femoral neck bone mineral density T-scores.

Source	Sum of squares	df	Mean square	F	*P* value
Phase angle tertile	33.829	2	16.915	18.274	<0.001
Age group	7.728	1	7.728	8.349	0.004
Interaction between phase angle and age	10.273	2	5.137	5.549	0.005
Error	149.026	161	0.926		
Total	617.836	167			

df, degrees of freedom.

Supplementary Table S3. Post hoc tests of mean femoral neck bone mineral density T-score across phase angle tertiles by age group performed using Tukey's honestly significant difference.

Age group	Difference in levels	Difference in means	Difference in SE	95% CI	*P* value
Younger	Tertile 1 – Tertile 2	-0.418	0.238	(-0.983, 0.147)	0.189
	Tertile 1 – Tertile 3	-0.492	0.226	(-1.029, 0.045)	0.080
	Tertile 2 – Tertile 3	-0.074	0.226	(-0.611, 0.463)	0.943
Elderly	Tertile 1 – Tertile 2	-1.145	0.288	(-1.839, -0.452)	0.001

Tertile 1 – Tertile 3	-1.776	0.314	(-2.531, -1.02)	0.000
Tertile 2 – Tertile 3	-0.630	0.314	(-1.386, 0.125)	0.120

CI, confidence interval; SE, standard error.
